# Commentary Monitoring Blood Chemistry Data in the Japanese National Health and Nutrition Survey: A Method and Its Significance

**DOI:** 10.2188/jea.JE20120178

**Published:** 2013-01-05

**Authors:** Kazuhiko Kotani

**Affiliations:** 1 Department of Clinical Laboratory Medicine, Jichi Medical University, Shimotsuke, Tochigi, Japan; 2 National Heart, Lung and Blood Institute, National Institutes of Health, Bethesda, MD, USA

**Keywords:** biochemical data, total error, national survey

This issue of the Journal includes the report entitled *Revised System to Evaluate Measurement of Blood Chemistry Data From the Japanese National Health and Nutrition Survey and Prefectural Health and Nutrition Surveys* by Nakamura and colleagues.^1^ Using quality control data recorded during 1999–2010, the authors calculated the total error (TE) for 14 blood chemistry items used in the Japanese National Health and Nutrition Survey (J-NHNS) and Prefectural Health and Nutrition Surveys.^1^ TE levels were used to evaluate whether blood chemistry data in the 2011 surveys were acceptable, and the authors determined that the respective items were all within acceptable and/or borderline limits.^1^ The authors propose that TE levels should be used to monitor blood chemistry data in the future.^1^

Errors in blood biochemical measurements are unavoidable,^2^ even though modern laboratories can considerably reduce such errors and clinical laboratory professionals carefully control data to minimize errors as part of their daily work. In addition, new analytical methods are continually being developed. In fact, the introduction of new analytical methods can affect the accuracy of blood chemistry items (eg, high-density lipoprotein cholesterol, creatinine).^1^ However, healthcare providers are not always aware that some errors are unavoidable and that analytical methods are sometimes changed. The work of Nakamura et al^1^ provides a rationale and method for comparing year-by-year blood chemistry data in the J-NHNS, a nationwide database of health-related information. If blood chemistry items show non-acceptable TE values in a certain year, researchers must take considerable care in sequentially comparing item data in that year with those in other years.

TE has been used in the National Cholesterol Education Program in the United States.^3^ It is calculated based on the accuracy and precision of blood chemistry items^1^ and is considered a good approach for assessing the analytical performance of such items, since the accuracy (%bias) and precision (reproducibility) are simultaneously and comprehensively considered. The concept of TE can obviate the complex considerations required for separately specifying accuracy and precision in practical applications.^3^ In addition, the tentative TE levels for the J-NHNS were previously reported by the first author of this report.^4^ Compared with the previous report,^4^ the current report^1^ determines TE values for various blood chemistry items, with an increased number of blood chemistry items measured in the J-NHNS. Many of the present blood chemistry items have not been extensively studied, and therefore the system proposed in the current report may serve as a reference for other countries.

Nationwide blood biochemistry data are a basic resource that can be used to describe national health status and, in turn, create new strategies for maintaining population health. For year-by-year observation of health status, various methodologies must be considered. The method proposed by Nakamura et al^1^ will contribute to observation of long-term changes in blood chemistry data in national surveys. Subsequent monitoring of blood biochemistry data in the near future is now a realistic expectation.
